# Effect of sex and age on traumatic brain injury: a geographical comparative study

**DOI:** 10.1186/s13690-017-0211-y

**Published:** 2017-10-09

**Authors:** Raaj Kishore Biswas, Enamul Kabir, Rachel King

**Affiliations:** 1 Faculty of Health, Engineering and Sciences (HES), University of Southern Queensland, Darling Heights, Toowoomba, QLD 4350 Australia; 2 School of Agricultural, Computational and Environmental Sciences, University of Southern Queensland, Darling Heights, Toowoomba, QLD 4350 Australia

**Keywords:** Public health, Glasgow outcome scale, Health geography, Bangladesh, Ordinal outcome scale

## Abstract

**Background:**

Traumatic brain injury (TBI) is a much researched topic in medical health, which requires additional studies to understand various effects of demographic and geographic factors that can assist in developing the most effective treatments. Thousands of people of different ages are suffering from lifelong disabilities, either mild or severe, from TBI and the number is increasing. This study aims to increase our understanding of the effect of sex and age by applying five different statistical methods to evaluate the effect of these covariates on two independent TBI data sets representing patients from different geographical cohorts. A primary data was collected from Bangladesh and it was compared with CRASH (Corticosteroid Randomisation after Significant Head Injury) data, representing various countries around the world.

**Methods:**

The outcome variable for TBI considered in this paper is Glasgow Outcome Scale, which is a four point scale. It was converted to a binary outcome scale for fitting of Fisher’s exact test, a test of proportions and a binary linear model. For analyzing ordinal outcomes, the proportional odds model and the sliding dichotomy model were fitted. As the sample size of the Bangladeshi data set was small, parametric bootstrapping was applied for the consistency of results.

**Results:**

Females were the worse sufferers of TBI compared to men, according to CRASH data set. The old (aged above 58 years) followed by adults (age 25 to 58) were the most vulnerable victims. Interaction effects concluded that old women tended to endure the worst outcomes of TBI. This conclusion came from the CRASH data set representing the world in general, whereas such effects were not present in the Bangladesh data set. Additional application of parametric bootstrapping for the smaller Bangladesh data set did not result into any significant outcome.

**Conclusion:**

The effect of gender and age could be stronger in some countries than others which is driving the significance in CRASH and was not found in Bangladesh. It reflects the necessity of incorporating geographic patterns as well as demographic features of patients while developing treatments and designing clinical trials.

## Background

Identification of effective treatments for traumatic brain injury (TBI) has been the focus of much medical research in recent years [1]. Improved understanding of the role of sex and age will contribute to the development of more patient and geographic specific treatments. TBI is defined as an alteration in brain function, or other evidence of changed brain pathology, caused by an external force to the brain [2]. Alteration in brain function generally means any period of loss or a decreased level of consciousness (LOC). Although not all blows or jolts to the head result in TBI [3]. TBI is one of the most common forms of severe injury with a high death toll or life-long disabilities seen among patients. Among the injuries that occur due to TBI, the recorded deaths number more than 50,000 yearly in the USA [4]. Each year approximately 370,000 new cases of TBI are hospitalized in USA [5] and the figure is more than 100,000 for Europe [6]. Young people are the most common sufferers of TBI, resulting in long term disabilities which, in addition to the personal toll, affects both the work force and economy [7]. Expenditure on TBI related costs in the USA alone is estimated to be $17 billion per year [8]. Severe and moderate forms of TBI, accidental or self-inflicted, are a major health and socioeconomic problem throughout the world [9]. Sex has been shown to be a key differentiating factor in many areas of medical research, and is often found to significantly interact with other predictor variables [10]. In the case of TBI patients it has been shown that the fatality rates are significantly higher for females than for males. Kraus et al. (2000) concluded that the mortality rate of women compared to men suffering from TBI was 1.28 times higher on average [11]. Moreover it was also found that even when death was not considered, women were 1.57 times more likely to suffer from post-traumatic symptoms than men. Klauber et al. (1981) also showed that fatality was higher for women compared to men in different age groups [12]. Even after one year of injury, severity of symptoms were more evident in women [13]. Several studies have also shown that women are prone to suffer more from TBI within one to three months of the traumatic incident [14–16]. On the other hand, the frequency of accidents leading to brain injury are more common in men than in women [17]. Males are more likely to be in recurrent accidents [11]; for example, motor cycle accidents are one of the most frequent causes of TBI in men [18]. Another common contributing factor leading to TBI incidents is the consumption of alcohol, which is more regularly consumed by men in general [19]. In summary, Farace et al. (2000) showed that in 17 out of 20 studies, which analyzed the effect of sex on TBI outcomes women suffered worse overall from TBI events [20].

It is generally accepted that the effect of diseases and injuries gradually worsens as age increases. TBI has shown to conform to this trend and different age groups are considered an important covariate in TBI studies [21]. Children have shown better recovery rates than older patients due to their greater degree of neuroplasticity [22]. Susman et al. (2002) found that mortality after a TBI event was approximately 24% in the elderly population while only 12.8% in other age groups [23]. Falls are the most common causes of brain injury in older patients and assaults or accidents in younger cohorts [24]. Even when older patients suffer comparatively minor head injuries and their overall injuries are seemingly less severe than non-elderly patients, they still have slower recovery rates and tend to experience more distress. Gómez et al. (2000) showed that the chance of an adverse outcome was 10 times higher for patients over 35 years of age compared to those aged between 15 to 25 years [21]. The large effect that age can play in long term outcomes for patients was shown in a study by Heiskanen et al. (1970), who found that less than 30% of patients aged 50 years or more went back to their former work, while more than 70% of patients under 20 years were able to go back to a normal life after their treatment [25]. Additionally, elderly patients had lower recovery rates than the young, while the young were more frequent sufferers of TBI [26].

Among older patient groups there is often a greater chance of co-morbidity occurring along with the primary disease or injury [27], as shown in studies of cardiovascular diseases [28], depression [29] and Alzheimer [30]. A positive association between depression and age following a TBI has been identified [31], indicating that older patients are more likely to suffer episodes of depression after head injury than younger patients. Guralnik and Jack (1996) identified a significant interaction effect between sex and age, with older women more likely to have higher prevalence of co-morbidity in contrast to older men [27]. Interestingly, sex difference had no impact on outcome scales if TBI was sustained by children, however for middle aged women TBI outcomes were significantly worse than for middle aged men. More elderly people suffered from TBI than middle aged people, however the difference between outcomes for elderly men and women (aged above 45) was less pronounced [19].

The aim of this paper is to investigate both the independent and interaction effects of age and sex on the TBI outcome scale, commonly known as the Glasgow Outcome Scale (GOS), (described in the following section). This study aims to increase our understanding of the effect of sex and age by applying five different statistical methods to evaluate the effect of these covariates on two independent TBI data sets representing patients from different geographical cohorts and find out the most vulnerable age-sex group for TBI.

## Method

### Data description

The first of the two data sets used in this study was the CRASH (Corticosteroid Randomisation after Significant Head Injury) data set which is comprised of data collected from TBI patients in a range of countries worldwide. The second data set, measuring the same variables as CRASH, was collected from TBI patients in Bangladesh, which was not one of the countries included in CRASH. These two data sets represent very different populations, with different levels of variation among a range of demographic and socio-economic variables not measured or included as covariates in this study. Of interest, was whether the different data sets suggested differences in the effects of age and sex on TBI for these different populations.

The CRASH data set was the result of a randomized controlled trial (ISRCTN74459797) [32]. This large trial was one of the most recent randomized trials monitoring the effect of corticosteroids on head injury and provided a large data set; which was suitable for applying different statistical models on the ordinal outcome variable measuring changes in patients’ post-treatment outcomes. Only the patients who were at least 16 years old and were observed whilst in hospital (in the absence of sedation) to have a GCS of 14 or less, and were within 8 hours of injury, were eligible for the trial entry. The CRASH collaboration includes data from various countries of Europe, Africa, South America, Asia and Oceania. The total number of patients in this data set was 10,800. Early results from the original CRASH study were published on 8 October 2004 (Lancet 2004;364:1321-28) and the 6-month follow-up results in May 2005 (Lancet 2005;365:1957-59) [33]. After removing cases with missing values, the final sample size was 7236. Results presented in this paper based on this data set do not distinguish between contributing countries. This Bangladesh data were collected from National Institute of Neuro Sciences and Hospital, Sher-e-Bangla Nagar, Dhaka in Bangladesh. The data is comprised of all brain injury patients treated in the hospital from May to September in 2015 with a total sample size 151. Patient information was collected from the hospital data base and cross checked with the resident physicians.

Glasgow Outcome Scale (GOS) was the outcome variable in both data sets. GOS is an ordinal variable commonly used to measure a recovering patients’ neurological responses after some form of treatment, [34]. Although the TBI treatments applied in the CRASH and Bangladesh data sets were different, they were each applied consistently across sex and age groups within the data sets. There have been some recent adjustments to the GOS scale within the medical community; however, the general format from worst to best outcome scales are Death (D), Vegetative State (VS), Severe Disability (SD), Moderate Disability (MD) and Good Recovery (GR). In this study Death (D) and Vegetative State (VS) were merged into a single category (named Vegetative State (VS)) because the sample size of deaths in the data sets was small. The independent variables were sex and age. The grouping criteria of age has evolved over generations of research and varies due to differing research aims [35]. The following age groupings are commonly accepted and they have been utilized in this study: ‘old’ (greater than 59 years), ‘adult’ (in between 25 and 58 years), and ‘young’ (aging 0 to 24 years). A separate category with patients aged below 15 was not created as their proportion in either data set was very small.

### Statistical methods

Frequencies for each level of the independent variables were calculated for each data set to provide a clear description of the data distributions. A cross tabulation of sex (two categories) and age (three categories) distribution with the GOS outcome variable (four categories) were also calculated. Five statistical models were applied as there is currently no single model that is considered the most robust approach when analyzing the ordinal outcomes of clinical trials [1, 9, 36, 37]. For the binary outcome analysis the GR & MD categories of the GOS scale were merged as favorable outcome and SD & VS were considered as unfavorable outcome. These binary levels of the outcome variable were created to analyze the effects of the covariates by applying Fisher’s exact test, test of proportions and linear logistic regression model. To analyze the four point ordinal outcome scale of GOS, the proportional odds model and sliding dichotomy model were applied. All of the tests assessed the probability of a favorable outcome over a non-favorable or less favorable outcome which was consistently defined as the reference group. The multinomial regression model was not considered in this study as it does not provide one unique odds ratio for each category unlike other models. All statistical analyses were performed in R (version3.2.3).

All of the statistical methods were applied to both data sets and results were compared. As the primary data set from Bangladesh was small, the analysis was performed a second time implementing parametric bootstrapping with 1000 replications to attain more precision.

### Binary outcome analysis

For assessing the significance of frequency distributions within a two-way contingency table, the common approach is to apply a chi-square goodness of fit test. However, this approach is only valid when expected frequency within cells is large [38]. Fisher’s exact test, developed by R.A. Fisher [39], was applied to the collected data in Bangladesh due to insufficient expected values. The test is also valid for large samples as well allowing its application on comparatively bigger CRASH trial as well.

The test of proportions was applied to analyze the null hypothesis that the proportion of ‘favorable outcome’ results (probabilities of success) in several groups are similar [40]. It is an alternate to the Fisher’s exact test and was applied here to consolidate the results from Fisher’s test. Fisher’s exact test and the test of proportions are appropriate methods only when the explanatory factor is also binary (e.g. sex).

The conventional binary linear logistic regression, or logit regression, first developed by D.R. Cox [41], is a popular model to analyze dichotomous forms of outcome variables. The logistic model is favored for its mathematical flexibility as well as clinically meaningful interpretation [42]. The linear logistic model is defined by Eq. 1,

1 $$ \boldsymbol{logit}\boldsymbol{\pi}_{\boldsymbol{i}} \boldsymbol{=} \boldsymbol{\log}\frac{\boldsymbol{\pi}_{\boldsymbol{i}}}{\boldsymbol{1}\boldsymbol{-}\boldsymbol{\pi}_{\boldsymbol{i}}} \boldsymbol{=} \boldsymbol{X}^{\boldsymbol{T}}\boldsymbol{\beta}  $$

where ***x***_***i***_ is a vector measurement corresponding to covariates and dummy variables corresponding to factor levels of the *X* covariate matrix and ***β*** is the parameter vector [43]. This model, referred in Eq. 1, is very widely used for analyzing data involving binary or binomial responses with several explanatory variables. It accommodates explanatory variables with more than two categories (e.g Age), providing a powerful technique analogous to multiple regression and ANOVA for continuous responses. The *glm* in R *MASS* Package was applied to fit this model.

### Ordinal outcome analysis

The first method used to analyze the ordinal form of the GOS outcome variable was the proportional odds model. Naïve dichotomization of the full ordinal scale leads to loss of information and efficiency, when analyzing the outcomes. The proportional odds model is a popular choice for analyzing the full range of ordinal outcomes and avoiding the need for arbitrary dichotomization [44]. Where a random variable be ***Y*** with ***J*** categories and ***π***_***1***_,***π***_***2***_ ***,…,*** ***π***_***J***_ denote the respective probabilities, with ***π***_***1***_ ***+*** ***π***_***2***_ ***+⋯+*** ***π***_***J***_ ***=*** ***1***. The cumulative logit model is defined by Eq. 2,

2 $$ {} \boldsymbol{\log}\frac{\boldsymbol{\pi}_{\boldsymbol{1}}\boldsymbol{+... +} \boldsymbol{\pi}_{\boldsymbol{j}}}{\boldsymbol{\pi}_{\boldsymbol{j+1}} \boldsymbol{+... +} \boldsymbol{\pi}_{\boldsymbol{J}}} \boldsymbol{=} \boldsymbol{x}_{\boldsymbol{j}}^{\boldsymbol{T}}\boldsymbol{\beta}_{\boldsymbol{j}} \boldsymbol{=} \boldsymbol{\beta}_{\boldsymbol{0j}} \boldsymbol{+} \boldsymbol{\beta}_{\boldsymbol{1}}\boldsymbol{x}_{\boldsymbol{1}} \boldsymbol{+... +} \boldsymbol{\beta}_{\boldsymbol{p-1}}\boldsymbol{x}_{\boldsymbol{p-1}}  $$

where the ***x***’s are the covariates and the ***β***’s are the unknown parameters with the intercept term ***β***_***0j***_ (if exists) [43]. This model has a crucial odds assumption which claims that, the effects of the covariates ***x***_***1***_ ***,…,*** ***x***_***p−1***_ are same for all categories of the logarithmic outcome scale, resulting in a constant ***β*** value. The method estimates a common odds ratio over all possible cut-offs of the outcome scale for a given change in category within the covariates [45]. Package *Polr* from R was applied to fit this model.

**Fig. 1:**
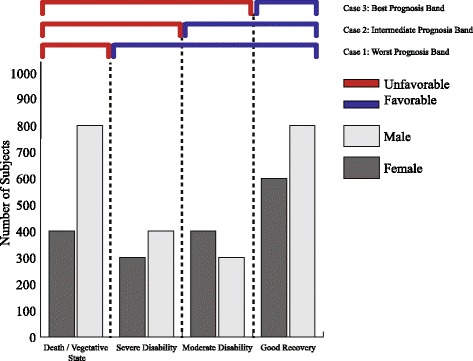
Sliding Dichotomy model: binary bands are created from ordinal scales

The sliding dichotomy model is a comparatively newer approach developed for clinical trials, particularly for TBI research [36]. This method is an improved version of the conventional logistic regression model. This model is assumed to provide the highest possible power and most robust results compared to the traditional methods in a number of scenarios. However, these scenarios are mostly limited to those cases when the probability of favorable outcomes is high [46]. Cases do exist where the fixed dichotomy and the proportional odds model performed better than the sliding dichotomy model [37]. Prior to analysis, outcome bands or successive dichotomous groups are created by segregating the fitted values (prognostic scores) from a binary logistic model [37]. Each band, displayed in Fig. 1, has its own reformed version of dichotomous ‘favorable’ and ‘unfavorable’ outcomes by combining a different subset of sequential outcomes from the original ordinal scale. The binary outcomes from all the bands are then compiled together and the traditional logistic regression model applied again to fit with available covariates, which were sex and age groups here. The *glm* in R *MASS* Package was applied to attain the fitted values as well as to analyze the complied favorable and unfavorable outcomes.

## Results and discussion

Table 1 shows the frequency of female patients was proportionately higher in the Bangladeshi data (32.5*%*,*n*=102) compared to the CRASH data (19*%*,*n*=7236). Based on the age group categories chosen for this analysis, there were relatively more ‘adults’ in the CRASH data (59*%*,*n*=4286) compared to the Bangladeshi data (40*%*,*n*=60). In the Bangladeshi data, patients in the ‘old’ and ‘adult’ categories together represented the majority of the sample (75.5*%*,*n*=114); however, ’old’ category only comprised of 8.5% of the data in CRASH where the majority of patients were consisted of ‘adult’ and ‘young’.

**Table 1:** Frequency distribution of sex and age in both data sets

Covariates	Levels	Bangladeshi data (151)	CRASH data (7236)
Gender	Female	102(67.5%)	5856 (80.9%)
	Male	49 (32.5%)	1380 (19.1%)
Age Groups	Old (>59)	54 (35.8%)	616 (8.5%)
	Adult (25 ∼58)	60 (39.7%)	4286 (59.2%)
	Young (0 ∼25)	37 (24.5%)	2334 (32.2%)

### Sex

Table 2 displays the frequency distribution of patients by sex for each of the two forms of outcome variable, binary and ordinal.

**Table 2:** Distribution of sex by GOS in binary and four point ordinal form

	Bangladeshi data (151)		CRASH data (7236)	
Outcome scales	Male (% among male)	Female(% among female)	Male (% among male)	Female(% among female)
Favorable (GR & MD)	77 (75.5%)	37 (75.5%)	4904 (83.7%)	1094 (79.3%)
Unfavorable (SD & VS)	25 (24.5%)	12 (24.5%)	952 (16.3%)	286 (20.7%)
GR	55 (53.9%)	23 (46.9%)	3511 (60%)	822 (59.6%)
MD	22 (21.6%)	14 (28.6%)	1393 (23.8%)	272 (19.7%)
SD	06 (5.9%)	07 (14.3%)	747 (12.8%)	209 (15.1%)
VS	19 (18.2%)	05 (10.2%)	205 (3.5%)	77 (5.6%)

Based on the binary outcome data (Table 2), the Bangladeshi data appeared to have a higher proportion of unfavorable outcomes for both males and females (24.5*%* & 24.5*%*, *n*=25 & 12) compared to the CRASH data (16.3*%* & 20*%*, *n*=952 & 286). The proportion of males and females did not vary much between the data sets for the GR and MD categories of the four point GOS ordinal scale; however, the SD and VS proportional differences were comparatively high.

Fisher’s exact test and the test of proportions did not show any significant difference (*P*>0.05) between sexes on the binary GOS measure (Table 3) for the primary data collected from Bangladesh. In contrast, the difference between sexes was found to be highly significant (*p*<0.001) in the CRASH data for these same statistical methods. The odds indicate that females were 26% more likely to have unfavorable outcomes after treatment for TBI than men. In general terms, women were more prone to suffer unfavorable outcomes due to TBI than men demonstrated by global data. In addition, Table 3 presents the results of the binary logistic regression for dichotomous form of GOS and the proportional odds modeling and sliding dichotomy method for the four point ordinal GOS. The data from Bangladesh did not show any significant difference (*P*>0.05) between males and females, neither in considering the binary GOS nor the four scale ordinal GOS. These results were consistent both with and without parametric bootstrapping; indicating that the smaller sample size of the Bangladeshi data (when compared to CRASH) was not influencing the method performance or results. However, a significant difference was detected between males and females in the CRASH data for all the three methods (*P*<0.001). The odds of unfavorable outcomes for women were 0.74 (0.64∼0.86), 0.91 (0.81∼1.03) and 0.97 (0.82∼1.14) in the logistic regression, the proportional odds model and the sliding dichotomy model respectively. These values agreed with previous tests and indicated that women have higher chance of having more suffrage from TBI, varying from 03 to 26%, compared to men.

**Table 3:** Statistical models on GOS by sex

Tests		Bangladeshi data	Bootstrap of Bangladeshi data	CRASH data
Fisher’s exact test	*P*-value	1		<0.001
	CI	0.427 ∼ 2.441		0.639 ∼ 0.863
	Odds	1.001		0.743
Test of proportions	*P*-value	0.99		<0.001
	CI	-0.174 ∼ 0.173		-0.074 ∼ -0.023
Binary logistic model	*P*-value	0.998	0.998	<0.001
	CI	0.453 ∼ 2.211	0.453 ∼ 2.211	0.641 ∼ 0.861
	Odds	1.001	1.001	0.743
Proportional odds model	CI	0.477 ∼ 1.682	0.477 ∼ 1.682	0.813 ∼ 1.025
	Odds	0.896	0.896	0.913
Sliding dichotomy model	*P*-value	0.841	0.841	0.688
	CI	0.441 ∼ 2.735	0.441 ∼ 2.736	0.816 ∼ 1.144
	Odds	1.098	1.098	0.966

The reference level for sex was ‘male’

### Age

The data sets did not vary much in the proportion of different categories of age (Table 4). The ‘young’ category is comparatively higher in proportion in the CRASH data compared to the Bangladeshi data. The GR and MD groups had the higher proportion of samples for both Bangladeshi data and CRASH data.

**Table 4:** Distribution of GOS (as binary outcome) over age

	Bangladeshi data (151)			CRASH data (7236)		
Outcome scales	Old (% among Old)	Adult (% among adult)	Young (% among Young)	Old (% among Old)	Adult (% among adult)	Young (% among Young)
Favourable (GR & MD)	12 (22.2%)	17 (28.3%)	8 (21.6%)	197 (32%)	781 (18.2%)	260 (11.1%)
Unfavourable (SD & VS)	42 (77.8%)	43 (71.7%)	29 (78.4%)	419 (68%)	35.05 (81.8%)	2074(88.9%)
GR	26 (48.1%)	29 (48.3%)	23 (62.2%)	313 (50.8%)	2444 (54%)	1576 (67.5%)
MD	16 (29.6%)	14 (23.3%)	6 (16.2%)	106 (17.2%)	1061 (24.8%)	498 (21.3%)
SD	5 (9.3%)	6 (10%)	2 (5.4%)	138 (22.4%)	610 (14.2%)	208 (8.9%)
VS	7 (13%)	11 (18.3%)	6 (16.2%)	59 (9.6%)	171 (04%)	52 (2.2%)

The binary logistic model, the proportional odds model and the sliding dichotomy model were applied to fit age groups with GOS. Both the binary regression and proportional odds model agreed that the ‘adult’ and the ‘young’ groups were significantly different (*P*<0.001) from the ‘old’ in CRASH data (Table 5). However, no significance was found in the sliding dichotomy model for the age groups in either data sets. According to the binary logistic model, applied in CRASH data, adults were 2.1 times and youths were 3.8 times more likely to have favorable outcomes in TBI compared to the olds. The Proportional odds model determined the likeliness of favorable outcomes in case of adults and youths were 1.6 and 2.5 times higher as TBI patients than olds respectively. These gave a summary stating olds were the worst victims of TBI. A contrasting conclusion was attained from the Bangladeshi data, both for normal and bootstrapping procedures. There were no mentionable differences between the age groups over the TBI outcomes in Bangladesh for the three models.

**Table 5:** Statistical tests on age groups vs GOS

Tests		Bangladeshi data		Bootstrap of Bangladeshi data		CRASH data	
		Adult	Young	Adult	Young	Adult	Young
Binary logistic model	*P*-value	0.455	0.946	0.455	0.946	<0.001	<0.001
	CI	0.308 ∼ 1.695	0.376 ∼ 2.849	0.308 ∼ 1.695	0.376 ∼ 2.849	1.751 ∼ 2.542	3.031 ∼ 4.640
	Odds	0.723	1.034	0.723	1.036	2.110	3.751
Proportional odds model	CI	0.452 ∼ 1.761	0.650 ∼ 3.331	0.452 ∼ 1.761	0.649 ∼ 3.331	1.331 ∼ 1.849	0.855 ∼ 7.299
	Odds	0.892	1.471	0.892	1.471	1.569	2.498
Sliding dichotomy model	*P*-value	0.815	0.955	0.815	0.955	0.513	0.332
	CI	0.338 ∼ 2.349	0.334 ∼ 3.19	0.337 ∼ 2.349	0.334 ∼ 3.198	0.714 ∼ 1.18	0.673 ∼ 1.143
	Odds	0.891	1.0333	0.891	1.0333	0.919	0.877

The reference level for age group was ‘old’

### Sex and age

The demography of age groups and sex in each data set in cross-frequency distribution (Table 6) showed the proportions of adults varied between the data sets. The ‘old’ group and the ‘young’ group appeared to be similarly proportioned in both male and female. The percentage of female patients in both data sets were comparatively lower than males.

**Table 6:** Cross table of sex and age in both data sets

	Bangladeshi data (151)		CRASH data (7236)	
Sex	Male (% among the age group)	Female (% among the age group)	Male (% among the age group)	Female (% among the age group)
Age groups				
Old (>59)	37 (68.5%)	17 (31.5%)	411 (66.7%)	205 (33.3%)
Adult (25 ∼58)	39 (65%)	21 (35%)	3534 (82.5%)	752 (17.5%)
Young (15 ∼25)	26 (70.3%)	11 (29.7%)	1911 (81.9%)	423 (18.1%)

Sex, age and their interaction effect were fitted in the same model for the binary logistic regression, proportional odds model and the sliding dichotomy method. The results obtained from the Bangladeshi primary data set are displayed in Table 7 and CRASH data in Table 8. None of the tests displayed any significant covariates or interactions in the Bangladeshi data. In contrast, CRASH data showed the significance of sex (reference group ‘male’) and the two age groups: ‘adult’ and ‘young’ (reference group ‘old’) in the logistic regression model and the proportional odds model. The interaction effect between ‘sex (female) and age (adult)’ along with ‘sex (female) and age(young)’ were found to be significant (*p* *v* *a* *l* *u* *e*<0.05). As the interaction effects were detected only in the CRASH data set, further analysis were conducted to assess the significance of interaction effects in that data set, displayed in Table 9. The interaction models were fitted for the binary regression model and the proportional odds model only, as the sliding dichotomy model failed to show any significant interactions.

**Table 7:** Statistical tests on age groups and sex with interactions for Bangladesh data

Bangladeshi data						
Tests		Sex (Female)	Adult	Young	Sex*Adult	Sex*Young
Binary logistic model	*P*-value	0.876	0.681	0.627	0.774	0.289
	CI	0.229 ∼ 3.517	0.276 ∼ 2.315	0.233 ∼ 2.407	0.128 ∼ 4.613	0.300 ∼ 56.222
	Odds	0.897	0.800	0.749	0.769	4.109
Proportional odds model	CI	5.0218 ∼ 0.063	0.273 ∼ 1.972	0.350 ∼ 2.528	0.408 ∼ 6.831	0.649 ∼ 24.912
	Odds	0.563	0.734	0.941	1.669	4.020
Sliding dichotomy model	*P*-value	0.516	0.913	0.716	0.568	0.554
	CI	0.323 ∼ 9.477	0.334 ∼ 3.402	0.334 ∼ 4.931	0.061 ∼ 4.653	0.038 ∼ 5.795
	Odds	1.750	1.067	1.283	0.531	0.468

The reference level for sex was ‘male’ and age group was ‘old’

**Table 8:** Statistical tests on age groups and sex with interactions for CRASH data

CRASH data						
Tests		Sex (Female)	Adult	Young	Sex*Adult	Sex*Young
Binary logistic model	*P*-value	<0.001	<0.001	<0.001	0.018	0.024
	CI	0.372 ∼ 0.752	1.367 ∼ 2.181	2.339 ∼ 3.935	1.089 ∼ 2.447	1.076 ∼ 2.819
	Odds	0.529	1.727	3.034	1.633	1.742
Proportional odds model	CI	0.415 ∼ 0.796	1.073 ∼ 1.598	1.671 ∼ 2.545	1.214 ∼ 2.496	1.252 ∼ 2.759
	Odds	0.574	1.309	2.062	1.741	1.858
Sliding dichotomy model	*P*-value	0.615	0.803	0.697	0.594	0.37
	CI	0.682 ∼ 1.905	0.712 ∼ 1.302	0.686 ∼ 1.286	0.489 ∼ 1.505	0.423 ∼ 1.378
	Odds	1.141	0.962	0.939	0.858	0.763

The reference level for sex was ‘male’ and age groups was ‘old’

**Table 9:** Interaction effects for CRASH data

CRASH data						
Tests		Male*Adult	Male*Young	Female*Old	Female*Adult	Female*Young
Binary logistic model	*P*-value	<0.001	<0.001	<0.001	<0.001	<0.001
	CI	1.367 ∼ 2.181	2.339 ∼ 3.935	0.372 ∼ 0.752	1.126 ∼ 1.975	1.937 ∼ 4.031
	Odds	1.727	3.034	0.529	1.490	2.794
Proportional odds model	CI	1.073 ∼ 1.598	1.671 ∼ 2.545	0.415 ∼ 0.796	1.035 ∼ 1.656	1.668 ∼ 2.904
	Odds	1.309	2.062	0.574	1.309	2.201

The reference level considered here was Male*Old

Both the tests showed adult male, adult female, young male and young female groups face less severity in TBI compared to old males, however this was not true for old females. Additionally, the binary regression model showed that old females had a 47% lesser chance of favorable outcome than old men. Both adult and young females suffered more than adult and young men. However, adult women and young women faced 1.5 and 2.8 times better outcomes than old men respectively. The odds of favorable outcome were 1.7 and 3.0 for adult and young men respectively compared to old men showing the faster recovery by youths and adults in contrast with the older patients. Although the difference in odds between adult men and adult women were comparatively closer in the proportional odds model, the gap between young males and females were evident in both models but in opposite directions. CRASH, the multi country data which sampled a wider and more varied population, showed a significant interaction of age and sex on TBI. This suggests that the effect of gender and age could be stronger in some countries than others, which is driving the significance in CRASH that was not found in Bangladesh.

The worst victims of TBI, sequentially in descending order were old females, old men, adult women, adult men and then the young. This conclusion was derived from the CRASH data, which was a multi-country data set. These effects were not evident in the data from Bangladesh. The sensitivity of the human brain is higher than other organs and therefore it is likely that effective treatment of brain injury will require greater patient specificity. Inclusion of additional covariates measuring other patient demographic features, as well as information about the type of accident that has resulted in a patient’s TBI, may improve understanding of why older females are suffering more than others. This paper highlights the necessity of incorporating geographic patterns as well as demographic features of patients while developing treatments and designing clinical trials.

## Conclusions

This paper aimed to understand how demographic features, particularly sex and age, affect treatments of TBI and furthermore if location variation contributes to differences in these effects All results consistently indicated that there was no significant difference in GOS as a measure of TBI by either sex or age groups in the Bangladeshi sample. Additionally, no interactions between sex or age categories were found to be computationally significant. A clear distinction was found between males and females within the international CRASH data set, with females generally having worse outcomes. Significant differences were also found between some age groups within this data, with elderly patients more likely to suffer negative outcomes than patients within the adult and young age categories. Interaction effects identified for this data indicated that old women appeared to show the worst outcomes followed by old men.

From the analysis of these two data sets it appears that while sex and age were not strong covariates based on the Bangladeshi data, they were both significantly associated with GOS outcomes in the CRASH data. This suggests that head injury in Bangladesh and/or the impact of demographic factors on outcomes in Bangladesh may be different, or less important, than these factors in the rest of the world. Country wise analysis of the CRASH data is needed to determine if these results are common to all or most contributing countries in the CRASH data set or whether these results are influenced by one or a few countries only. This was not possible in the current study as country of origin information was not included in the available CRASH data. Furthermore, age in a continuous scale might provide additional information in future studies. The analysis of more health and demographic variables such as previous disease history particularly neurological or psychiatric problems, immunity level, mental health status, marital status, and workplace stress would help to clarify the recovery profile of patients.

## Abbreviations

CRASH: Corticosteroid randomisation after significant head injury
GOS: Glasgow outcome scales
GR: Good recovery
MD: Moderate disability
SD: Severe disability
TBI: Traumatic brain injury
VS: Vegetative State
